# P-194. Evolution of *Clostridioides* difficile Antimicrobial Susceptibility within the epidemic REA Group BI (RT027) and the endemic REA group Y (RT014/020)

**DOI:** 10.1093/ofid/ofae631.398

**Published:** 2025-01-29

**Authors:** Andrew M Skinner, Laurica A Petrella, Adam K Cheknis, Jennifer Cadnum, Curtis Donskey, Larry K Kociolek, Matthew H Samore, Dale N Gerding, Charlesnika T Evans, Stuart Johnson

**Affiliations:** University of Utah, Salt Lake City, Utah; Edward Hines Jr VA Hospital, Hines, Illinois; Edward Hines Jr. VA Hospital, Hines, Illinois; Northeast Ohio VA Medical Center, Cleveland, Ohio; Cleveland VA Hospital, Cleveland, Ohio; Ann & Robert H. Lurie Children's Hospital of Chicago, Chicago, IL; University of Utah, Salt Lake City, Utah; Edward Hines, Jr. Veterans Affairs Hospital, Hines, Illinois; Northwestern University and VA, Hines, Illinois; Hines VA Hospital and Loyola University Medical Center, Hines, Illinois

## Abstract

**Background:**

Antimicrobial resistance in *Clostridioides difficile* (CD) has been a key contributor for the spread of specific endemic CD strains. Monitoring changes in antimicrobial minimum inhibitor concentration (MIC) over time could potentially predict future CD outbreaks. We determined the change in antimicrobial MIC over 40 years for 10 critical antibiotics in one epidemic and one endemic CD strain groups.

**Figure:**
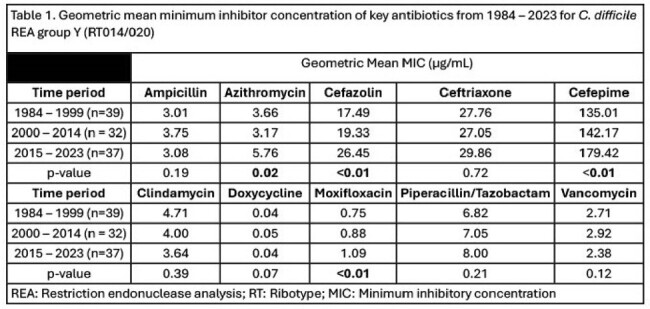

**Methods:**

Antimicrobial agar dilution was performed on 193 clinical CD isolates collected from 1984 – 2023 identified as the epidemic restriction endonuclease analysis (REA) group BI (RT027) or the endemic REA Y (RT014/020) group. Geometric mean MICs were compared for isolates collected between 1984 – 1999, 2000 – 2014, and 2015 – 2023 by Kruskal-Walis test. A generalized additive model was constructed for each antibiotic that had significant changes in the geometric mean MIC for each REA group.

**Figure 1.**
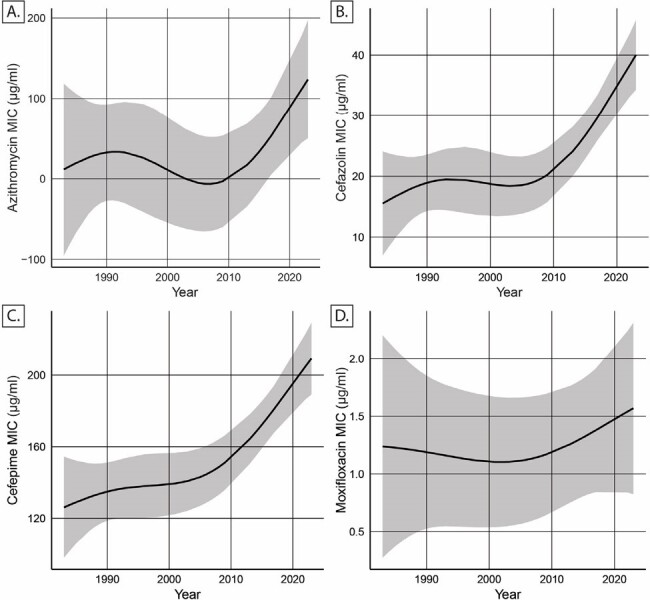
Change in key antibiotic MICs for isolates identified as REA group Y (RT014/020) from 1984 – 2023

Generalized additive model showing the change in:

A. C. difficile azithromycin MIC from 1984 – 2023

B. C. difficile cefazolin MIC from 1984 – 2023

C. C. difficile cefepime MIC from 1984 – 2023

D. C. difficile moxifloxacin MIC from 1984 – 2023

Gray Bar reflects 95% confidence interval

MIC: Minimum inhibitory concentration

REA: Restriction endonuclease analysis

RT: Ribotype

**Results:**

Among the endemic REA group Y isolates there were increases in the cefazolin and cefepime MICs from 17.49 µg/ml to 26.45 µg/ml (p< 0.01) and 135.01 µg/ml to 179.42 µg/ml (p< 0.01). (Table 1) The increase in the azithromycin, cefazolin, and cefepime MICs appears to be increasing since 2005 for REA group Y. (Figure 1) For the epidemic REA group BI isolates the ceftriaxone MIC increased from 20.16 µg/ml to 51.51 µg/ml (p< 0.01) and the cefepime MIC increased from 168.90 µg/ml to 256.00 µg/ml (p< 0.01), respectively. The azithromycin MIC peaked in the 2000-2014 period (733.43 µg/ml) but trended downward in the 2015 – 2023 period (322.54 µg/ml, (p< 0.01). The moxifloxacin MIC peaked in the 2000 – 2014 period (42.99 µg/ml) but then decreased in the 2015 - 23 period (16.00 µg/ml, p< 0.01). (Table 2) The ceftriaxone, cefepime, and piperacillin/tazobactam MIC increase appears to be constant over time for BI isolates. However, the moxifloxacin MIC has decreased while the azithromycin MIC has stabilized since 2005. (Figure 2)

**Figure:**
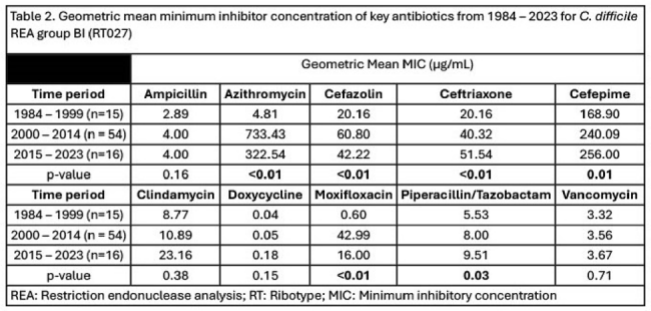

**Conclusion:**

CD MIC for cefazolin, ceftriaxone, and cefepime have increased in both REA group BI and REA group Y isolates over 40 years. However, moxifloxacin and azithromycin MICs are trending downward from their peak in the early 2000s for REA group BI. These data indicate that further monitoring and a greater understanding of cephalosporin resistance is prudent to understanding future outbreaks.

**Figure 2.**
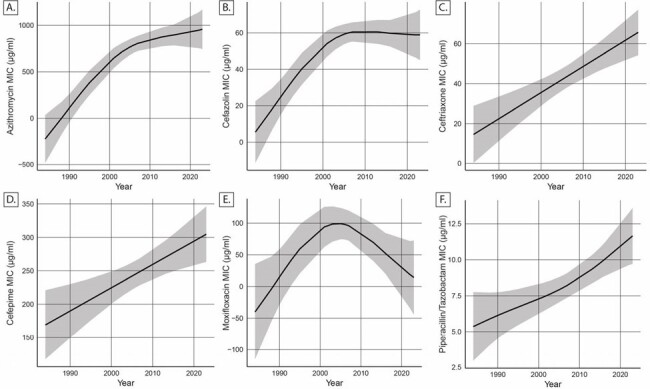
Change in key antibiotic MICs for isolates identified as REA group BI (RT027) from 1984 – 2023

Generalized additive model showing the change in:

A. Change in C. difficile azithromycin MIC from 1984 – 2023

B. Change in C. difficile cefazolin MIC from 1984 – 2023

C. Change in C. difficile ceftriaxone MIC from 1984 – 2023

D. Change in C. difficile cefepime MIC from 1984 - 2023

E. Change in C. difficile moxifloxacin MIC from 1984 – 2023

F. Change in C. difficile piperacillin/tazobactam MIC from 1984 – 2023

Gray Bar reflects 95% confidence interval

MIC: Minimum inhibitory concentration

REA: Restriction endonuclease analysis

RT: Ribotype

**Disclosures:**

**Andrew M. Skinner, MD**, BioK plus: Advisor/Consultant|Ferring Pharmaceuticals: Advisor/Consultant|Recursion pharmaceutical: Advisor/Consultant **Curtis Donskey, MD**, Clorox: Grant/Research Support|Pfizer: Grant/Research Support **Larry K. Kociolek, MD, MSCI**, Merck: Grant/Research Support **Dale N. Gerding, MD**, AstraZeneca: Advisor/Consultant|Destiny Pharma: Advisor/Consultant|Destiny Pharma: Licensed IP to Destiny|Sebela: Advisor/Consultant|Sebela: Licensed IP **Stuart Johnson, M.D.**, Acurx Pharmaceuticals: Advisor/Consultant|Bio-K Plus International: Advisor/Consultant|Ferring Phamraceuticals: Advisor/Consultant

